# The ChinaMAP reference panel for the accurate genotype imputation in Chinese populations

**DOI:** 10.1038/s41422-021-00564-z

**Published:** 2021-09-06

**Authors:** Lin Li, Peide Huang, Xiaohui Sun, Siyu Wang, Min Xu, Sha Liu, Zhimin Feng, Qing Zhang, Xiaoji Wang, Xiaole Zheng, Mengyao Dai, Yufang Bi, Guang Ning, Yanan Cao, Weiqing Wang

**Affiliations:** 1 Department of Endocrine and Metabolic Diseases, Shanghai Institute of Endocrine and Metabolic Diseases, National Clinical Research Centre for Metabolic Diseases, Key Laboratory for Endocrine and Metabolic Diseases of the National Health Commission, State Key Laboratory of Medical Genomics, Ruijin Hospital, Shanghai Jiao Tong University School of Medicine, Shanghai, China; 2 National Research Center for Translational Medicine, National Key Scientific Infrastructure for Translational Medicine, Shanghai Jiao Tong University, Shanghai, China; 3 BGI, BGI-Shenzhen, Shenzhen, Guangdong China; 4 SJTU-BGI Innovation Research Center, Shanghai, China

**Keywords:** Bioinformatics, Genomic analysis

Dear Editor,

The genotype imputation is an efficient and pivotal approach to estimate the unobserved genotypes in the genomic data from the single nucleotide polymorphism (SNP) genotyping arrays or whole-genome sequencing (WGS). The fine mapping of variants in the genome-wide association study (GWAS) could be critically improved by the imputation with an optimal reference panel based on the population-specific haplotypes.^1^ The significant progresses of imputation were recently achieved by large high-resolution reference panels, such as the 1KGP3 (1000 Genomes Project Phase 3, *n* = 2504),^2^ the UK10K (10,000 UK Genome Sequences, *n* = 3781),^3^ the HRC (Haplotype Reference Consortium, *n* = 32,470),^4^ TOPMed (Trans-Omics for Precision Medicine, *n* = 97,256)^5^ and the GAsP (The GenomeAsia 100 K Project, *n* = 1654)^6^ (Supplementary information, Table S1). The multi-ethnic 1KGP3 reference panel is most commonly used for the genetic studies of Asian populations. However, the European ancestry-dominant reference panels exhibited poor performance in the genotype imputation for Chinese and other East Asian populations.^7–9^ The number of well-imputed variants and imputation accuracy depend on the characteristics of the reference panel dataset, including the sample size, sequencing data quality and population composition. The limited Chinese samples restrict the imputation quality with current reference panels in Chinese population studies. Additionally, the insufficient sequencing depth of construction datasets could undermine the detection capacity of rare variants for the imputation. Therefore, a population-specific reference panel constructed by a large-scale, in-depth WGS dataset of Chinese populations could be essential for the accurate and comprehensive imputation of the genotyping array and sequencing data from Chinese individuals.^9,10^

To achieve high-quality imputation for Chinese genomic datasets, we constructed a high-resolution and population-specific reference panel based on large-scale (10,155 unrelated Chinese individuals), in-depth (40.80×) WGS data from the China Metabolic Analytics Project (ChinaMAP).^11^ Compared to the previous reference panels with Chinese samples, the ChinaMAP reference panel showed significant advances in sample size and sequencing depth (Supplementary information, Table S1). The ChinaMAP reference panel contains 59.01 M SNPs, including 44.03 M known SNPs and 14.98 M novel SNPs in comparison with the combination of the databases of TOPMed, gnomAD (Genome Aggregation Database), dbSNP (Single Nucleotide Polymorphism Database) and 1KGP3 (Supplementary information, Fig. S1a). The majority of novel SNPs (99.65%) were very rare (allele frequency (AF) ≤ 0.1%). The principal component analysis showed that the ChinaMAP reference panel was comprised of seven Chinese Han subpopulations and seven ethnic minorities (Supplementary information, Fig. S1b).^11^ Compared to the commonly used reference panels 1KGP3, HRC, GAsP and TOPMed, the ChinaMAP reference panel contains 30.24 M specific SNPs (Fig. 1a), contributing to a more comprehensive imputation and more novel findings in the genetic studies of Chinese population. The ChinaMAP imputation server could be accessed on the ChinaMAP website (www.mbiobank.com) for genotype imputation and download of result and variant database.

**Fig. 1:**
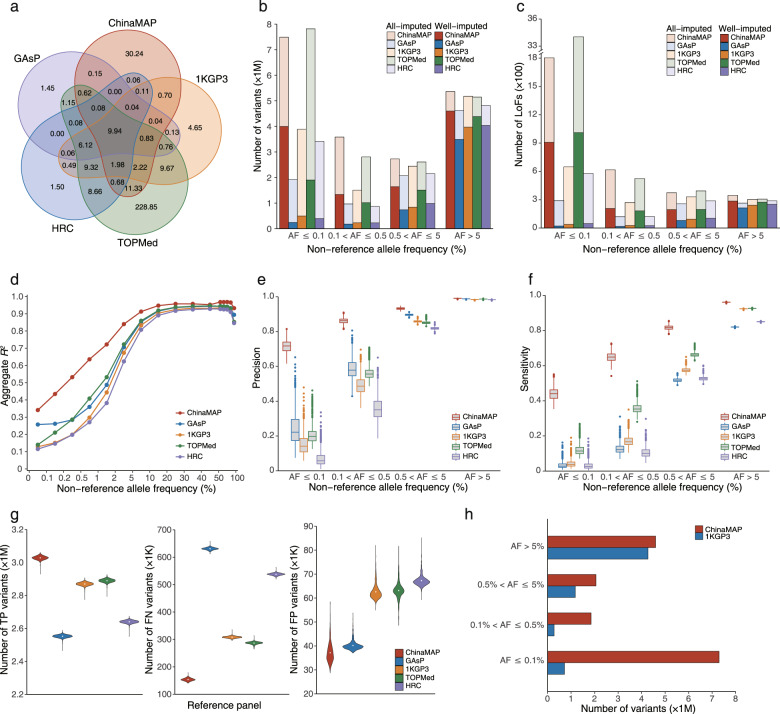
The imputation performance of the ChinaMAP reference panel. **a** The number of all specific and shared variants (× 1 M) in the ChinaMAP, 1KGP3, HRC, TOPMed and GAsP reference panels. **b**, **c** The imputed and well-imputed (estimated *R*^*2*^ ≥ 0.8) variants (**b**) and LoFs (**c**) with different allele frequencies generated by the imputation of mimic UK Biobank array data from a WGS dataset (*n* = 794) with different reference panels. **d** The comparison of imputation accuracy between the ChinaMAP and other reference panels by aggregate *R*^*2*^ values. **e**, **f** The imputation precision (**e**) and sensitivity (**f**) of the ChinaMAP, GAsP, 1KGP3, TOPMed and HRC reference panels. **g** The number and distribution of true positive (TP), false negative (FN) and false positive (FP) variants generated by the imputation of mimic UK Biobank array data with different reference panels in the 794 WGS samples. **h** The well-imputed variants with different allele frequencies generated by the imputation of MAPCGA array data (*n* = 4775) with the ChinaMAP or 1KGP3 reference panels.

To evaluate the performance of the ChinaMAP reference panel, we compared the ChinaMAP with four commonly used reference panels for genotype imputation. We used an independent WGS dataset (40× depth, 36,450,184 SNPs) of 794 Chinese individuals from the ChinaMAP phase 2 to mimic a typical imputation analysis. The genotypes of 460,481 variants on the UK Biobank Axiom Array and 517,745 variants on the Infinium Asian Screening Array (ASA) were extracted after filtering the monomorphic variants and imputing the masked genotypes with different reference panels, respectively. The estimated squared correlation (*R*^2^) was calculated for each imputed variant to evaluate the imputation quality. The results showed that the ChinaMAP reference panel exhibited the best performance for the number of well-imputed variants (estimated *R*^2^ ≥ 0.8, 11.58 M for UK Biobank and 11.72 M for ASA) and the highest quality for the shared imputed variants (9.51 M, mean *R*^2^ = 0.70 for UK Biobank; 9.48 M, mean *R*^2^ = 0.68 for ASA) among the five reference panels (Supplementary information, Table S2). The coverage of variants (AF ≥ 0.5%) in the ChinaMAP database could increase to 76% by the imputation with the ChinaMAP reference panel. Moreover, the ChinaMAP reference panel significantly increased the numbers of well-imputed low-frequency (0.5% < AF ≤ 5%), rare (0.1% < AF ≤ 0.5%) and very rare (AF ≤ 0.1%) variants and improved the well-imputed common variants (AF > 5%) compared to other reference panels (Fig. 1b; Supplementary information, Table S3 and Fig. S2a). The ChinaMAP and TOPMed reference panels could impute far more variants than the 1KGP3, HRC and GAsP reference panels in general. Additionally, we compared the well-imputed loss-of-function variants (LoFs), which are potentially clinically relevant and of particular interest in genetic investigations. The results showed that the ChinaMAP and TOPMed reference panels could obtain much more well-imputed LoFs than other panels (Fig. 1c; Supplementary information, Table S4 and Fig. S2b).

Furthermore, the squared correlation coefficient (aggregate *R*^2^) between the imputed allele dosages and the masked genotypes in the independent WGS dataset was calculated with each reference panel to compare the imputation accuracy in different allele frequencies. As expected, the ChinaMAP reference panel had the highest imputation accuracy overall (Fig. 1d; Supplementary information, Fig. S2c). In the comparison of imputation results and WGS genotypes, the ChinaMAP reference panel showed the best imputation precision (positive predictive value) and sensitivity (true positive rate) across all the frequency bins among the five reference panels (Fig. 1e, f; Supplementary information, Fig. S2d, e). The ChinaMAP reference panel imputed the largest number of true positive variants, and the lowest number of false positive and false negative variants in the comparison among the five reference panels (Fig. 1g; Supplementary information, Figs. S2f, S3). In particular, the genotyping results of very rare variants from low-coverage sequencing regions or microarray data were more likely to contain false positive and false negative signals. Our data indicated that the imputation of very rare LoFs (AF ≤ 0.1%) by the ChinaMAP and TOPMed reference panels might need further validation (Supplementary information, Fig. S4). The 1KGP3, HRC and GAsP reference panels may be unreliable for the imputation of rare and very rare LoFs (AF ≤ 0.5%) due to lack of true positive signals. Taken together, the population-specific ChinaMAP reference panel exhibited significant improvement in the accurate imputation for genotyping data of Chinese individuals compared to other reference panels.

To further assess the imputation efficiency of the ChinaMAP reference panel in GWAS analysis, we performed an imputation of genotyping array data of 4775 individuals from the ChinaMAP phase 2. The genotyping of 728 K variants was performed using the Chinese genome array (MAPCGA) designed based on the ChinaMAP database.^11^ The imputation using the ChinaMAP and 1KGP3 reference panels generated 15.8 M and 6.45 M well-imputed variants, respectively. The coverage of variants with AF ≥ 0.5% and AF ≥ 5% in the ChinaMAP database could be increased to 83.36% and 88.17% by the ChinaMAP reference panel (Fig. 1h; Supplementary information, Table S5). The markedly increased 9.35 M well-imputed variants could lead to promising and better results in the following GWAS or polygenic risk score analyses. In addition, the ChinaMAP reference panel also obtained the largest number of well-imputed variants in the imputation of 728 K variants on the MAPCGA array using the WGS dataset of 794 individuals (Supplementary information, Table S6).

In summary, the ChinaMAP reference panel was constructed for accurate and comprehensive imputation based on the large, high-coverage WGS dataset. The population-specific ChinaMAP reference panel exhibited significant superiorities in the well-imputed number, imputation accuracy, precision and sensitivity compared to the previous reference panels in the evaluations using mimic or actual genotyping data. The ChinaMAP imputation server (www.mbiobank.com) may provide an optimal imputation method for genetic studies of Chinese and East Asian populations.

## Supplementary information

Supplementary information

## Data Availability

The ChinaMAP imputation server can be accessed on the ChinaMAP browser (www.mBiobank.com). The researchers can upload the unphased genotypes to the imputation server and receive the phased and imputed genotypes in return following the instructions on the website. The ChinaMAP variant database and the reference panel site files can be downloaded following the regulation of the Human Genetic Resources Administration of China (HGRAC).
