# Supplementary material for: The ChinaMAP reference panel for the accurate genotype imputation in Chinese populations
Source: Cell Res. 2021 Sep 6;31(12):1308–10. doi: 10.1038/s41422-021-00564-z (PMC8648815; doi:10.1038/s41422-021-00564-z)
Supplement: Supplementary file 1 — Supplementary information [file 41422_2021_564_MOESM1_ESM.pdf]

SUPPLEMENTARY INFORMATION

The ChinaMAP reference panel for the accurate genotype imputation in Chinese populations

Lin Li[1,2], Peide Huang[3], Xiaohui Sun[2], Siyu Wang[2], Min Xu[1], Sha Liu[1], Zhimin Feng[1], Qing Zhang[1,2], Xiaoji Wang[4], Xiaole Zheng[3], Mengyao Dai[2], Yufang Bi[1], Guang Ning[1,2], Yanan Cao[1,2] and Weiqing Wang[1]

[1]Department of Endocrine and Metabolic Diseases, Shanghai Institute of Endocrine and Metabolic Diseases, National Clinical Research Centre for Metabolic Diseases, Key Laboratory for Endocrine and Metabolic Diseases of the National Health Commission, State Key Laboratory of Medical Genomics, Ruijin Hospital, Shanghai Jiao Tong University School of Medicine, Shanghai 200025, China. [2]National Research Center for Translational Medicine, National Key Scientific Infrastructure for Translational Medicine, Shanghai Jiao Tong University, Shanghai 200240, China. [3]BGI, BGI-Shenzhen, Shenzhen 518083, China. [4]SJTU-BGI Innovation Research Center, Shanghai 200240, China.

These authors contributed equally: Lin Li, Peide Huang, Xiaohui Sun, Siyu Wang

Correspondence: Yanan Cao (caoyanan@vip.sina.com) or Weiqing Wang (wqingw61@163.com)

Materials and Methods

DNA Samples

Genomic DNA was obtained from the metabolic biobank of the National Clinical Research Centre for Metabolic Diseases, Shanghai Clinical Center for Endocrine and Metabolic Diseases in Ruijin Hospital, Shanghai Jiao Tong University School of Medicine. Informed consent was obtained from all study participants. All the protocols were approved by the Ruijin Hospital Ethics Committee, Shanghai Jiao Tong University School of Medicine.

Construction of the ChinaMAP reference panel

The ChinaMAP reference panel was constructed based on the ChinaMAP phase 1 dataset (136,745,826 variants in 10,588 individuals)[11] using the following approaches (Supplementary information, Fig. S5). Firstly, we performed the IBD (identity-by-descent) analysis by using the PLINK[12] software. The 10,155 samples with a PI_HAT (the proportion of IBD between two individuals) value ≤ 0.05 were considered un-related samples and reserved for subsequent analyses. Secondly, we reserved all the variants with AC (variant allele count) > 1 (n = 59,611,800) and singleton variants (AC = 1, n = 3,924,016) which were included in commonly used microarrays. The following criteria filtered the samples with reserved variants: (1)

exclude samples with missing calling rate > 0.05; (2) exclude variants with missing calling rate > 0.05. Finally, a set of 59,010,860 SNPs from 10,155 individuals were reserved. The Shapeit4[13] software was used to rephase the genotype calls.

Principal component analysis

PCA was performed as described in our former study.[11] Briefly, the autosomal bi-allelic SNPs were selected according to the following criteria: (1) minor allele frequency (MAF) ≥ 1%; (2) genotyping rate ≥ 90%; (3) Hardy-Weinberg- Equilibrium (HWE) P > 0.000001; (4) removing one SNP from each pair with r-squared ≥ 0.5 (in windows of 50 SNPs with steps of 5 SNPs). Finally, 1,460,832 selected SNPs were used for PCA by using PLINK[12] (v1.9) and EIGENSOFT[14,15] (v7.2.1).

The ChinaMAP imputation server

The ChinaMAP imputation server is utilizing the ChinaMAP reference panel to perform imputation analysis online. The haplotype phasing and genotypes imputation were performed using Eagle2 and Minimac4 software. The imputation pipeline includes the following steps: (1)

the vcf files are parsed by the identification of chromosomes and checked by requirements in each file; (2) the 20 Mb file chunks are created with quality control to exclude sites without genotype or A, T, C, G sites or duplicate sites; (3) the chunks are excluded if the number of variants in the reference panel < 3 or more than 20% variants are not included in the reference panel; (4) the phasing for each valid chunk is executed by the Eagle2 with the following script (chr2:1-20000000 for example): /path/eagle --noImpMissing --chrom 2 --bpStart 1 --bpEnd 20000000 --vcfRef reference_panel.chr2.phased.vcf.gz --vcfTarget chr2.1-20000000.vcf.gz --geneticMapFile genetic_map.hg38.txt --allowRefAltSwap --vcfOutFormat z --outputUnphased --outPrefix chr2.1-20000000.phased; (5) the imputation for each valid chunk is executed by the Minimac4 with following script (chr2:1-20000000 for example): /path/minimac4 --chr chr2 --start 1 --end 20000000 --minRatio 0.000001 --window 500000 --refhaps reference_panel.chr2.m3vcf.gz --haps chr2.1-20000000.phased.vcf.gz --noPhoneHome --allTypedSites --format GT, DS, GP --prefix chr2.1-20000000.impute; (6) all chunks of one chromosome are merged into one single vcf.gz file.

Evaluation of the imputation performance

The independent whole-genome sequencing (WGS) data of 794 samples from the ChinaMAP phase 2 were used to assess the imputation performance of the ChinaMAP and other reference panels. The genotypes of variants on the UK biobank Array, the Infinium ASA or the MAPCGA Array were extracted from the WGS data for imputation respectively. The haplotype phasing and genotypes imputation with the ChinaMAP reference panel was performed by the Eagle2[16] and Minimac4[17] software. The Eagle2 estimates the haplotype phase with the phased reference panel using a new and very fast HMM-based (Hidden Markov Model) algorithm. The Minimac4 is based on the computationally efficient implementation of MaCH algorithm for genotype imputation. The Michigan Imputation Server[17] was used to generate the imputation results with the GAsP, 1KGP3 and HRC reference panels. The TOPMed Imputation Server was used to generate the imputation results with the TOPMed reference panel.

The mean estimated R^2 values for each panel were calculated to evaluate the number of accurately imputed variants. The imputed variants with an estimated $R^2 \geq 0.8$ were defined as high-quality variants. The aggregate R^2 values, which were squared correlation between the imputation allele dosages and true genotype dosages, were also calculated for each reference panel. The imputation sensitivity is calculated by TP/(TP+FN) and the precision is calculated

by TP/(TP+FP). The true positive variant (TP) indicates the imputed SNP genotype is consistent with the WGS genotype. The false positive variant (FP) indicates the imputed SNP genotype is inconsistent with the WGS genotype. The false negative variant (FN) indicates the imputed reference genotype is inconsistent with the WGS genotype.

Evaluation of well-imputed LoFs

The imputation results from mimic array data with different reference panels and the variants called from the WGS data of 794 samples were annotated by the VEP[18] software. The variants of stop lost, start lost, splice acceptor variant, splice donor variant, stop gained, transcript ablation and frameshift were considered as putative LoFs in the VEP annotation results. The imputed LoFs with an estimated $R^2 \geq 0.8$ were defined as well-imputed LoFs. The genotypes of imputed LoFs were compared to the WGS genotypes to analyze the number of true positive, false positive and false negative variants.

Imputation of genotyping array data

The genotyping array was performed on the Axiom GeneTitan platform (Thermo Fisher) with

the MAPCGA array (96-well plates). The data were analyzed by the Array Power Tools (APT v2.11.4, https://www.thermofisher.cn). The best practices genotyping analysis workflow was applied for QC and genotyping as described in the user guide of APT, including the following steps: 1. Generate the sample Dish QC (DQC) and QC call rate (QCCR). 2. Remove samples with DQC < 0.82 or QCCR < 0.97. 3. Remove the array plates with the QC passing samples less than 95% or with the average call rate of QC passing samples less than 98.5%. 4. All QC passing samples are co-clustered and assigned genotypes by the AxiomGT1 algorithm. 5. Identify the best performance probe set for the previously ungenotyped markers. 6. Generate the recommended variants list excluding not recommended markers.

A total of 4,775 samples in 50 array plates with 728K recommended markers passed the QC. The imputation of 722K autosomal markers from the genotyping data of 4,775 samples was performed by the ChinaMAP and 1KGP3 reference panels. The ChinaMAP phase 1 database was used as the reference to analyze the coverage of variants by the imputation results from the ChinaMAP and 1KGP3 reference panels.

Supplementary Figures

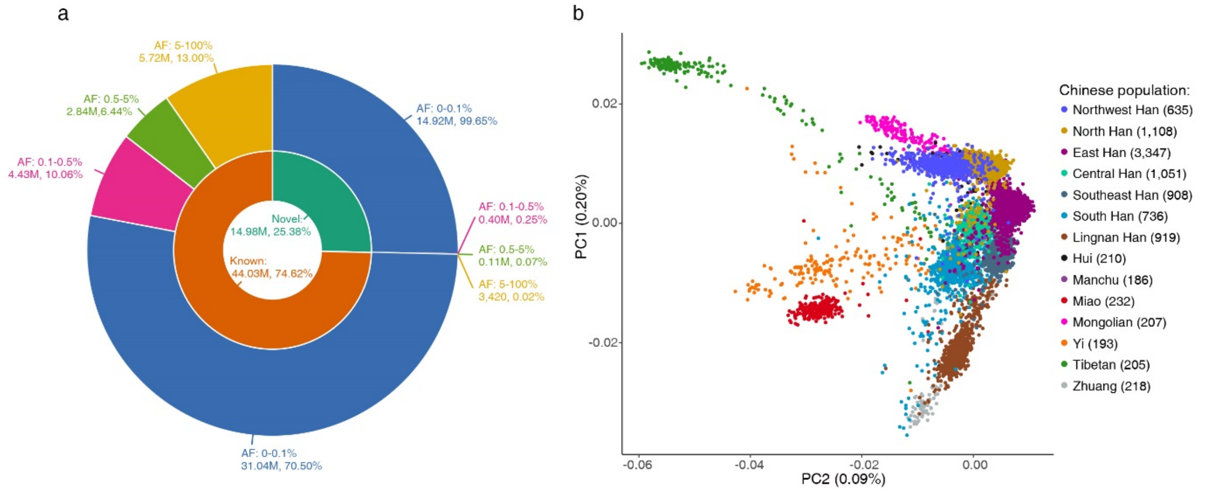

Fig. S1 The composition of the ChinaMAP reference panel. a The inner-circle showed the number and proportion of the novel (not included in the TOPMed freeze5, gnomAD v2.0.2, dbSNP v149 and 1KGP3 20130502 databases) and known (already exist in the TOPMed freeze5, gnomAD v2.0.2, dbSNP v149 or 1KGP3 20130502 databases) SNPs included in the ChinaMAP reference panel. The outer circle showed the number and proportion of SNPs with different AFs. **b** Principal component analysis of individuals in the ChinaMAP reference panel.

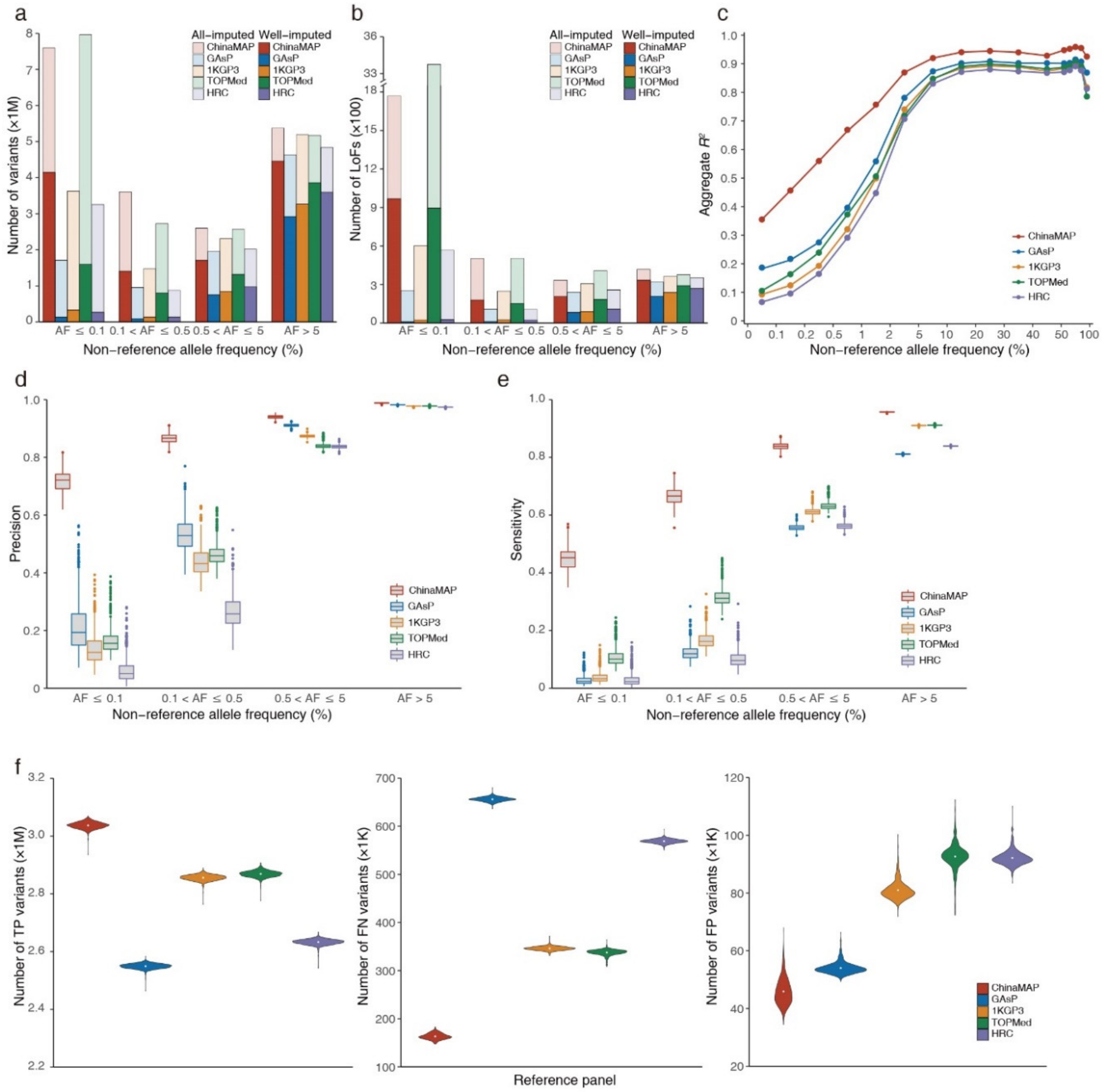

Fig. S2 The imputation performance of the ChinaMAP reference panel for the ASA array. **a, b** The imputed and well-imputed (estimated $R^2 \geq 0.8$) variants (**a**) and LoFs (**b**) with different allele frequencies generated by the imputation of mimic ASA data from a WGS dataset (n = 794) with different reference panels. **c** The comparison of imputation accuracy between the ChinaMAP and other reference panels by aggregate R^2 values. **d, e** The imputation precision (**d**) and sensitivity (**e**) of the ChinaMAP, GAsP, 1KGP3, TOPMed and HRC reference panels. **f** The number and distribution of true positive (TP), false negative (FN) and false positive (FP) variants generated by the imputation of mimic ASA array data with different reference panels in the 794 WGS samples.

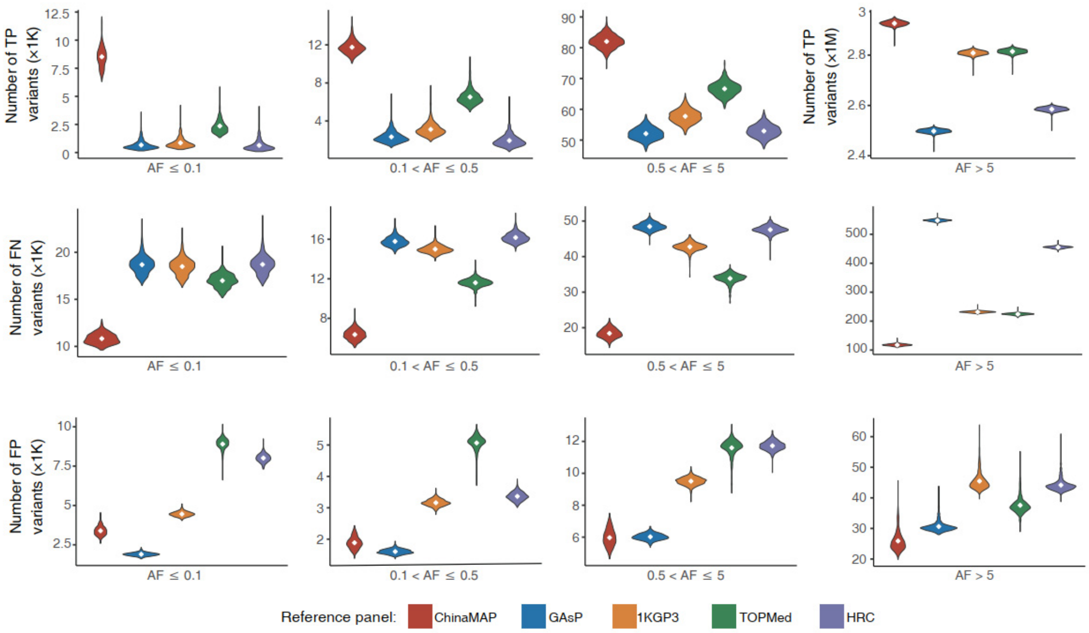

Fig. S3 Comparison of imputed variants and WGS genotypes. The number and distribution of true positive (TP), false negative (FN) and false positive (FP) variants generated by the imputation of mimic UK Biobank array data with different reference panels were analyzed in the 794 WGS samples.

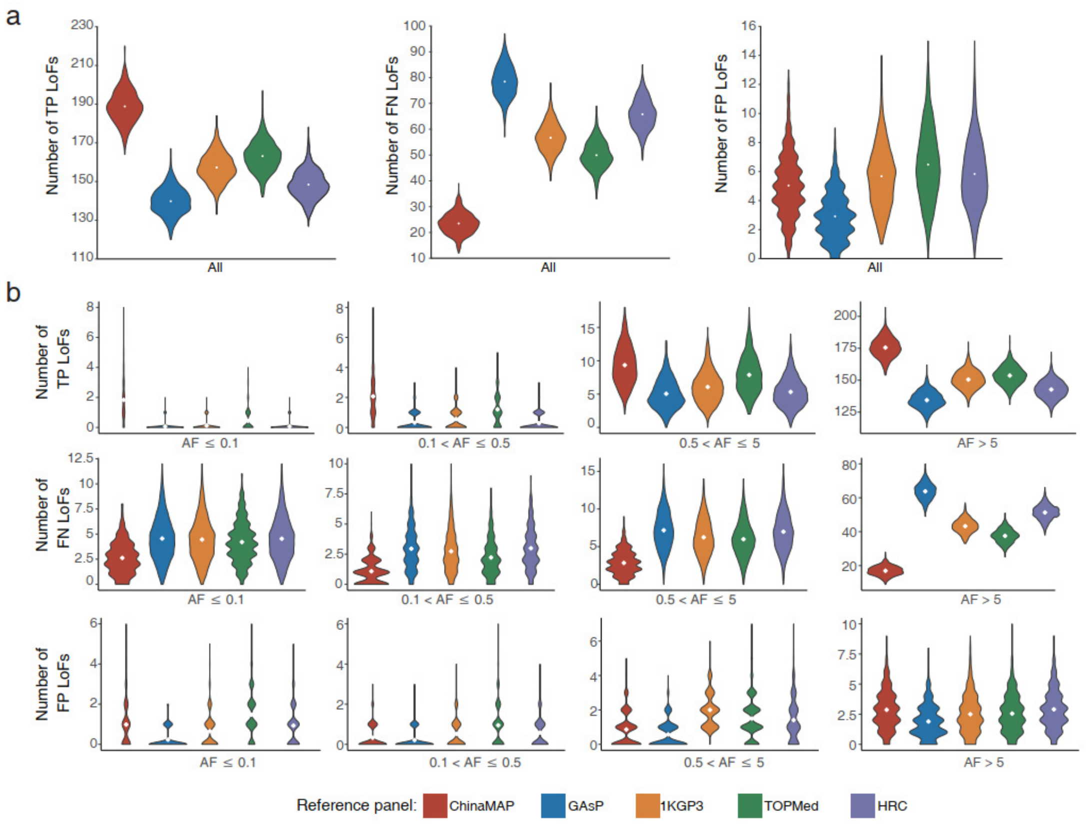

Fig. S4 Comparison of imputed LoFs and WGS genotypes. a The total number and distribution of true positive (TP), false negative (FN) and false positive (FP) LoFs generated by the imputation of mimic UK Biobank array data with different reference panels were analyzed in the 794 WGS samples. **b** The number and distribution of TP, FN and FP LoFs across different allele frequencies generated by the imputation of mimic UK Biobank array data with different reference panels were analyzed in the 794 WGS samples.

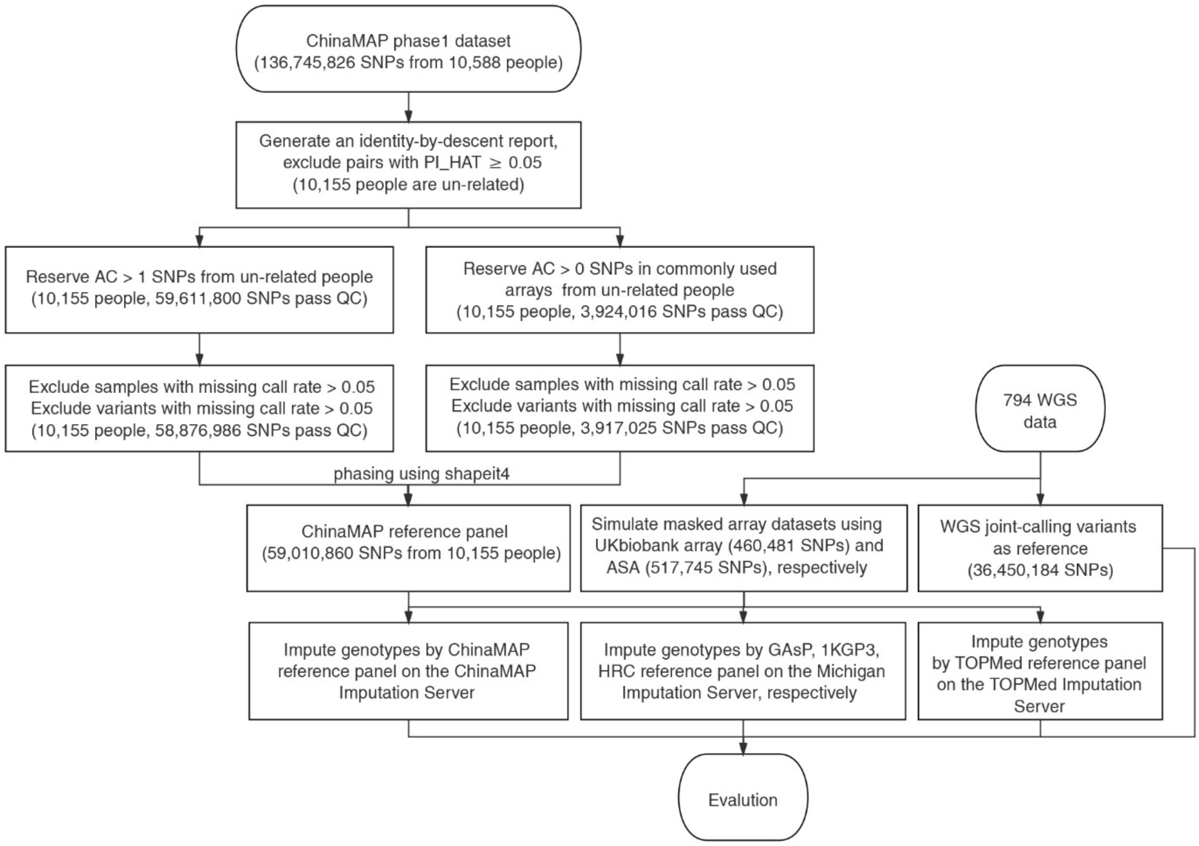

Fig. S5 The flowchart of construction and evaluation for the ChinaMAP reference panel.

Supplementary Tables

Table S1. The information of ChinaMAP and other reference panels.

Reference panel	Sequencing depth	Number of samples	Number of variants (M)	Ancestry distribution	Reference
**ChinaMAP**	40.8×	10,155	59.0	Chinese	11
**1KGP3**	7× WGS; 65× WES	2,504	49.1	Multiple ancestries	2
**UK10K**	7× WGS; 80× WES	3,781	42.0	European	3
**HRC**	4× – 8×	32,470	39.7	Predominant European	4
**TOPMed**	30×	97,256	308.1	Multi-ethnic	5
**GAsP**	30×	1,654	21.5	Asian	6
**1KJPN**	32.4×	1,070	21.2	Japanese	19
**NARD**	10× – 20×	1,781	22.9	Korean, Japanese, Chinese	20

Table S2. The statistics of imputation results of 5 different reference panels.

Array	Reference Panel	Number of variants	Imputed variants	Well-imputed variants	Shared imputed variants		Coverage of variants (AF > 0.5%) in the ChinaMAP database
					Number	Mean R^2	
UK Biobank	ChinaMAP	459,549	58,551,311	11,580,972	9,505,419	0.70	76.01%
	1KGP3	455,462	46,654,003	6,198,944	9,505,419	0.61	59.58%
	GAsP	440,958	21,052,632	4,660,306	9,505,419	0.59	52.73%
	HRC	457,421	38,663,584	5,653,394	9,505,419	0.67	61.96%
	TOPMed	455,811	291,798,799	9,473,368	9,505,419	0.69	72.01%
ASA	ChinaMAP	511,014	58,499,846	11,722,176	9,476,626	0.68	76.50%
	1KGP3	486,770	46,585,895	5,096,741	9,476,626	0.57	52.68%
	GAsP	481,416	21,012,174	3,895,105	9,476,626	0.55	47.60%
	HRC	487,984	38,629,121	4,975,852	9,476,626	0.64	57.97%
	TOPMed	374,198	291,770,336	8,144,348	9,476,626	0.65	63.72%

Table S3. The imputed and well-imputed variants generated by the imputation of mimic UK biobank array data from 794 WGS data with different reference panels*.

Reference Panel	Type	AF ≤ 0.1%	0.1% < AF ≤ 0.5%	0.5% < AF ≤ 5%	AF > 5%	ALL
	Imputed	7,488,047	3,583,808	2,729,423	5,365,872	19,167,150
ChinaMAP	Well-Imputed	3,997,823	1,337,773	1,643,573	4,601,803	11,580,972
	Well-Imputed rate	0.533894	0.373283	0.602169	0.857606	0.604209
	Imputed	1,915,886	968,410	2,072,701	4,620,948	9,577,945
GAsP	Well-Imputed	242,884	181,252	738,617	3,491,249	4,654,002
	Well-Imputed rate	0.126774	0.187165	0.356355	0.755527	0.485908
	Imputed	3,889,871	1,505,579	2,444,348	5,180,748	13,020,546
1KGP3	Well-Imputed	494,806	229,709	841,410	3,973,166	5,539,091
	Well-Imputed rate	0.127204	0.152572	0.344227	0.766910	0.425412
	Imputed	7,817,268	2,807,711	2,610,577	5,146,105	18,381,661
TOPMed	Well-Imputed	1,904,128	1,016,335	1,509,233	4,385,416	8,815,112
	Well-Imputed rate	0.243580	0.361980	0.578122	0.852182	0.479560
	Imputed	3,416,590	871,412	2,148,807	4,821,967	11,258,776
HRC	Well-Imputed	397,064	227,442	984,609	4,036,769	5,645,884
	Well-Imputed rate	0.116216	0.261004	0.458212	0.837162	0.501465

*The variants with AC = 0 have been removed.

Table S4. The imputed and well-imputed LoFs generated by the imputation of mimic UK biobank array data from 794 WGS data with different reference panels*.

Reference Panel	Type	AF ≤ 0.1%	0.1% < AF ≤ 0.5%	0.5% < AF ≤ 5%	AF > 5%	ALL
	Imputed	1,803	617	374	346	3,140
ChinaMAP	Well-Imputed	908	207	196	286	1,597
	Well-Imputed rate	0.503605	0.335494	0.524064	0.82659	0.508599

	Imputed	290	120	257	264	931
GAsP	Well-Imputed	22	18	80	214	334
	Well-Imputed rate	0.075862	0.15	0.311284	0.810606	0.358754
	Imputed	649	270	330	303	1,552
1KGP3	Well-Imputed	39	27	94	242	402
	Well-Imputed rate	0.060092	0.1	0.284848	0.79868	0.259021
	Imputed	3,403	524	393	306	4,626
TOPMed	Well-Imputed	1,011	182	197	274	1,664
	Well-Imputed rate	0.297091	0.347328	0.501272	0.895425	0.359706
	Imputed	578	122	288	289	1,277
HRC	Well-Imputed	47	25	103	252	427
	Well-Imputed rate	0.081315	0.204918	0.357639	0.871972	0.334377

*The variants with AC = 0 have been removed.

Table S5. The statistics of imputation results of 4,775 MAPCGA genotyping data by the ChinaMAP and 1KGP3 reference panels.

Reference Panel	AF	Number of variants	Well-imputed variants	Coverage of variants in the ChinaMAP	Coverage of variants in the ChinaMAP (AF > 0.5%)
ChinaMAP	AF ≤ 0.1%	38,344	7,296,329	5.94%	-
	0.1% < AF ≤ 0.5%	33,806	1,849,391	41.24%	-
	0.5% < AF ≤ 5%	127,333	2,059,628	75.27%	83.86%
	AF > 5%	512,738	4,594,796	88.17%	
1KGP3	AF ≤ 0.1%	38,344	720,396	0.61%	-
	0.1% < AF ≤ 0.5%	33,806	277,391	6.82%	-
	0.5% < AF ≤ 5%	127,333	1,178,265	44.93%	70.01%
	AF > 5%	512,738	4,271,463	82.59%	

Table S6. The imputed and well-imputed variants generated by the imputation of mimic MAPCGA array data from 794 WGS data with different reference panels*.

Reference Panel	Type	AF ≤ 0.1%	0.1% < AF ≤ 0.5%	0.5% < AF ≤ 5%	AF > 5%	ALL
ChinaMAP	Imputed	8,301,455	3,730,219	2,693,408	5,213,197	19,938,279
	Well-Imputed	5,129,436	1,950,729	2,146,834	4,655,492	13,882,491
	Well-Imputed rate	0.617896	0.522953	0.797070	0.893021	0.696273
GAsP	Imputed	188,1239	1,047,708	2,080,121	4,441,939	9,451,007
	Well-Imputed	219,396	158,026	973,067	3,730,205	5,080,694
	Well-Imputed rate	0.116623	0.150830	0.467793	0.839770	0.537582
1KGP3	Imputed	3,793,491	1,611,813	2,434,354	4,999,087	12,838,745
	Well-Imputed	497,355	224,364	1,078,885	4,137,324	5,937,928
	Well-Imputed rate	0.131107	0.139200	0.443191	0.827616	0.462501
TOPMed	Imputed	7,622,901	2,867,813	2,616,186	5,022,019	18,128,919
	Well-Imputed	1,977,374	1,103,726	1,689,239	4,390,348	9,160,687
	Well-Imputed rate	0.259399	0.384867	0.645688	0.874220	0.505308
HRC	Imputed	2,978,417	882,181	2,091,827	4,639,129	10,591,554
	Well-Imputed	374,137	200,059	1,193,451	4,079,350	5,846,997
	Well-Imputed rate	0.125616	0.226778	0.570530	0.879335	0.552043

*The variants with AC = 0 have been removed.

Supplementary References

12. Purcell, S. et al. PLINK: A tool set for whole-genome association and population-based linkage analyses. *Am. J. Hum. Genet.* **81**, 559-75 (2007).
13. Delaneau, O. et al. Accurate, scalable and integrative haplotype estimation. *Nat. Commun.* **10**, 5436 (2019).
14. Chang, C. C. et al. Second-generation PLINK: rising to the challenge of larger and richer datasets. *Gigascience* **4**, 7 (2015).
15. Price, A. L. et al. Principal components analysis corrects for stratification in genome-wide association studies. *Nat. Genet.* **38**, 904–909 (2006).
16. Loh, P. R. et al. Reference-based phasing using the Haplotype Reference Consortium panel. *Nat. Genet.* **48**, 1443–1448 (2016).
17. Das, S. et al. Next-generation genotype imputation service and methods. *Nat. Genet.* **48**, 1284–1287 (2016).
18. McLaren, W. et al. The Ensembl Variant Effect Predictor. *Genome Biol.* **17**, 122. (2016).
19. Nagasaki, M. et al. Rare variant discovery by deep whole-genome sequencing of 1,070 Japanese individuals. *Nat. Commun.* **6**, 1–13 (2015).
20. Yoo, S. K. et al. NARD: whole-genome reference panel of 1779 Northeast Asians improves imputation accuracy of rare and low-frequency variants. *Genome Med.* **11**, 64 (2019).
